# A designer cell culture insert with a nanofibrous membrane toward engineering an epithelial tissue model validated by cellular nanomechanics†

**DOI:** 10.1039/d1na00280e

**Published:** 2021-07-05

**Authors:** Prasoon Kumar, Dhaval Kedaria, Chinmaya Mahapatra, Monisha Mohandas, Kaushik Chatterjee

**Affiliations:** Department of Materials Engineering, Indian Institute of Science C.V. Raman Avenue Bangalore 560012 India kchatterjee@iisc.ac.in +91-80-22933408; Centre for BioSystems Science and Engineering, Indian Institute of Science C.V. Raman Avenue Bangalore 560012 India; Department of Biotechnology and Medical Engineering, National Institute of Technology Rourkela 769008 India; School of Chemical Engineering, Biomedical Institute for Convergence at SKKU (BICS), Sungkyunkwan University Suwon 16419 Republic of Korea

## Abstract

Engineered platforms for culturing cells of the skin and other epithelial tissues are useful for the regeneration and development of *in vitro* tissue models used in drug screening. Recapitulating the biomechanical behavior of the cells is one of the important hallmarks of successful tissue generation on these platforms. The biomechanical behavior of cells profoundly affects the physiological functions of the generated tissue. In this work, a designer nanofibrous cell culture insert (NCCI) device was developed, consisting of a free-hanging polymeric nanofibrous membrane. The free-hanging nanofibrous membrane has a well-tailored architecture, stiffness, and topography to better mimic the extracellular matrix of any soft tissue than conventional, flat tissue culture polystyrene (TCPS) surfaces. Human keratinocytes (HaCaT cells) cultured on the designer NCCIs exhibited a 3D tissue-like phenotype compared to the cells cultured on TCPS. Furthermore, the biomechanical characterization by bio-atomic force microscopy (Bio-AFM) revealed a markedly altered cellular morphology and stiffness of the cellular cytoplasm, nucleus, and cell–cell junctions. The nuclear and cytoplasmic moduli were reduced, while the stiffness of the cellular junctions was enhanced on the NCCI compared to cells on TCPS, which are indicative of the fluidic state and migratory phenotype on the NCCI. These observations were corroborated by immunostaining, which revealed enhanced cell–cell contact along with a higher expression of junction proteins and enhanced migration in a wound-healing assay. Taken together, these results underscore the role of the novel designer NCCI device as an *in vitro* platform for epithelial cells with several potential applications, including drug testing, disease modeling, and tissue regeneration.

## Abstract 2

A designer insert was fabricated with nanofibrous membrane as a culture platform for cells of the skin and other epithelial tissues that can be useful as *in vitro* tissue model and for drug screening applications.

## Graphical abstract

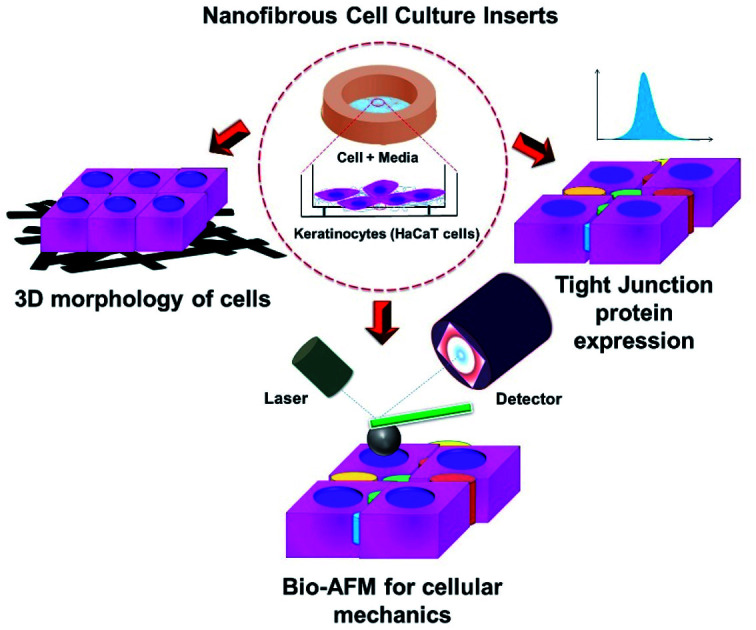

## 1. Introduction

The mechanical integrity of the cell and cell-to-cell junctions plays a vital role in defining and driving several biological functions of the human body and underlying changes in tissue development and disease formation. Some of these well-studied examples include the formation of organized monolayers of epithelial cells in the skin and lungs,^1,2^ selective transport of molecules across the intestinal epithelial lining of the gut, and glomerulus in the kidneys,^3,4^ epithelial to mesenchymal transition in cancer metastasis,^5^ mechanotransduction, and embryogenesis.^6^ Compromise of the cell-to-cell junctions due to acute wounds, mechanical trauma, surgical procedures, burns, and congenital diseases is associated with functional disorders of the kidneys, lungs, and small intestine, and progression of diseases such as cancer.^7,8^ Therefore, *in vitro* models that can faithfully recapitulate tissue barriers are invaluable for studying the molecular basis underlying the pathophysiology of tissues and could serve as platforms for testing novel therapeutics.

Several *in vitro* models using different technologies, such as three-dimensional (3D) tissue scaffolds and microfluidic devices, have been engineered for many tissues. Among them, 3D nanofibrous mats can effectively mimic the topography of the extracellular matrix (ECM) of human tissues.^9,10^ The role of the composition of the material, topography, and stiffness of nanofibers has been investigated to illustrate their effects on the attachment, spreading, proliferation, migration, and differentiation of cells.^11–14^ For instance, the aligned nanofibers of polycaprolactone and gelatin blend (PCL/Gel) supported the attachment, alignment, and differentiation of periodontal ligament stem cells for ligamentogenesis while down-regulating osteogenesis.^15^ The presence of nanopits with a tailored disorder of ±50 nm on polymethylmethacrylate (PMMA) facilitated the differentiation of skeletal stem cells towards osteogenic lineage as compared to substrates with flat and completely randomly oriented nanopits.^16^ It has been reported that a transition in cellular morphology from flat to less-spread cells occurs due to the rearrangement of cytoskeletal elements in response to the surface topography that provides focal adhesion sites.^17,18^ These cytoskeletal morphological changes are essential for biochemical mechanotransduction that plays a role in governing the phenotype of cells.^19^ Hence, the spherical morphology of cells offers better opportunities for cells to communicate and interact with the neighboring cells and underlying ECM. It is now well recognized that cells respond to changes in topography and the stiffness of the underlying substrate through cytoskeletal rearrangements.^20,21^ In contrast to the flatter morphology in a two-dimensional (2D) culture, the micro/nanotopographies on the nanofibers may further guide the cells to have an increased formation of cell-to-cell junctions such as tight junctions, and hence play a role in promoting the barrier properties of tissues.^19,22^

The formation of tight junctions in certain tissues results in selective transport of molecules through the cells and not through interstitial spaces. Investigations of the tight junction biology in epithelial cells such as keratinocytes are typically performed by culturing the cells in cell culture inserts. The cells form tight junctions, and the resultant control of transport across the cell monolayer is revealed by the transepithelial electrical resistance (TEER).^23,24^ However, owing to the flat morphology and high stiffness of the microporous polycarbonate membrane typically used in commercially available inserts, cells experience a 2D microenvironment. It has been demonstrated by Fung *et al.* that the elasticity of viable epithelial skin cells (HaCaT cells) with compromised desmosomal junctions cultured on coverslips changes using a bio-atomic force microscope (Bio-AFM).^25^ Furthermore, using a Bio-AFM, Matjaž *et al.* showed that the exposure of lamellar liquid crystals to keratinocytes at non-toxic concentrations affects the elastic modulus of the cells.^26^ Prior to or during these biomechanical studies, cells were either fixed or grown on a flat glass substrate where the substrate material hardly mimics the native microenvironment of keratinocytes. Thus, the supra-physiological stiffness of the substrate can alter the biomechanical properties of the keratinocytes. Torras *et al.* described the role of novel 3D culture devices based on bioprinting and photolithography that facilitate better epithelial tissue development for investigating tight junctions.^27^

Nanofibrous substrates are one of the most widely investigated scaffolds in tissue engineering. The nanofibrous substrates offer a surface topography and stiffness similar to that of native tissues, unlike the planar and rigid conventional 2D tissue culture polystyrene (TCPS) substrates. However, there are variations in the stiffness distribution on fibrous scaffolds due to the nanofibrous morphology, geometry, and loading boundary conditions. This may affect how the cells perceive the local micro-scale stiffness of the underlying scaffold and modify its response.^28–30^ It has been reported that the stiffness and micro-topologies of the underlying substrate affect the expression of junction proteins in stem cells, hence leading to enhanced transepithelial resistance.^31^ Although nanofibrous scaffolds faithfully recapitulate the 3D ECM microenvironment of the cells, the combined effect of micro-/nano-topography along with local stiffness of these fibrous scaffolds on the morphological properties of the cells is less understood.^32–34^

We hypothesize that the presence of nanoscale topography of a fibrous matrix in a free-hanging system will alter the cell morphology of keratinocytes to yield more mature tight junctions than the cells on TCPS. Therefore, in this work, we aimed to design and fabricate a designer cell culture insert device with a free-standing nanofibrous membrane for culturing human keratinocytes. The device not only offered micro/nanotopographies with low stiffness to the growing keratinocytes, an environment mimicking soft tissues, but also afforded biomechanical characterization of live cells using a Bio-AFM. We employed a Bio-AFM to characterize the changes in the mechanics of the cells and cell-to-cell junctions when cultured on the free-hanging mat compared to TCPS culture. The observed biomechanical properties were correlated with the changes in the cell organization and phenotypical changes. The potential of this platform to mimic the *in vivo* behavior of the epithelial cells is explored.

## 2. Materials and methods

### 2.1 Design and fabrication of the nanofiber-based cell culture inserts

The nanofiber-based cell culture insert (NCCI) was fabricated by combining electrospinning and 3D printing, as shown schematically in the ESI (Fig. S1A†). A computer-aided design (CAD) model of the NCCI was designed in SolidWorks and subsequently imported in a .STL format for 3D printing using a FabX3 machine (3Ding). The fused filament modeling (FFM)-based 3D printer used a poly(lactic acid) (PLA) filament (RedEx Technologies) for fabricating the structural parts of the NCCI after optimizing the printing parameters (Fig. S1A†). Thereafter, the base component (Fig. S1A†) was placed over a Whatman paper soiled with 0.01 M KCl solution (Fig. S1A†) to serve as the collector for electrospinning. A 12% (w/v) PCL (avg. *M*_n_ 80 000, Sigma Aldrich)/gelatin (Sigma Aldrich) blend solution (1 : 1 by wt) was prepared in 2,2,2-trifluoroethylene (TFE, Sigma Aldrich) with 50 μL of glacial acetic acid (S.D. Fine Chemicals Limited). The PCL/Gel solution was stirred overnight with a magnetic stirrer before electrospinning (ESPIN NANO V2, Physics Equipment Pvt. Ltd). The process parameters of electrospinning were optimized to operate at a voltage of 12 kV, a 12 cm distance between the collector and spinneret, a flow rate of the solution maintained by a syringe pump fixed at 0.5 mL h^−1^, a deposition time of 30 min, and a needle of 24G. Thereafter, a polydimethylsiloxane (PDMS, Sylgard 184, and Dow Corning) solution was prepared by mixing the base pre-polymer and curing agent in a 10 : 1 ratio. The PDMS solution was degassed and partially cured at 33 °C for 2 h to obtain transparent glue. This glue was used to bond the 3D printed part with the nanofiber mat, as shown in Fig. S1B.† The NCCI (Fig. S1B†) was finally obtained after fully curing the glue at room temperature (25 °C) for 12 h. The NCCIs were placed individually in 6 well plates, as shown in Fig. S1A(iii).†

### 2.2 Characterization of the fiber mat of the NCCI

A part of the nanofibrous mat was mounted on a copper stub and sputter-coated with gold before imaging by scanning electron microscopy (SEM, Zeiss Gemini with MonoCL) fitted with a secondary-electron detector (SE2) for characterization of the fibers. The fiber diameter and pore size were estimated by image processing in MATLAB 7.4 (MathWorks, Inc.). Additionally, the base components of the NCCI were also imaged with the SEM to characterize the fibers deposited. Fast-Fourier transformation (FFT) of the SEM images was performed to assess the extent of randomness in the alignment of the deposited nanofibers.

### 2.3 Cell culture

Immortalized human keratinocytes (HaCaT cells, Addexbio, San Diego, CA, USA) were cultured in Dulbecco's modified Eagle's medium-F12 (Lonza, Slough, UK) containing 4.5 g L^−1^ glucose, supplemented with 2 mM l-glutamine (Sigma, Dorset, UK), 100 IU per mL penicillin and 100 μg mL^−1^ streptomycin (Sigma, Dorset, UK), and 10% (v/v) fetal bovine serum (FBS, Fisher Scientific, Loughborough, UK). Cells were cultured in T25 flasks in an incubator at 37 °C with a 5% CO_2_ humidified atmosphere (Thermo, USA). The cell culture medium was replenished every 2 to 3 days, and the cells were passaged at 80 to 90% confluence using trypsin/EDTA (0.02% (w/v)) solution.

### 2.4 Cell studies on the NCCIs

The NCCIs were sterilized with ethylene oxide (Eto, An74i Anprolene gas sterilizer) and subsequently placed in a laminar flow hood for 5 h to remove any remnant Eto. The developed NCCIs were thoroughly washed in phosphate-buffered saline (PBS) and culture medium to remove any debris prior to seeding cells. 100 μL medium containing 3 × 10^5^ HaCaT cells was initially added to each well containing the insert. After 4 h, the well was filled with 2 mL of complete culture medium.

### 2.5 Characterization of cell morphology

Cell attachment and morphology on the insert was measured at 1 day after seeding cells. The cells were washed with 1× PBS, fixed in 4% formaldehyde for 15 min and subsequently permeabilized with 0.2% Triton X (Sigma-Aldrich). F-actin was stained with Alexa Fluor 546-conjugated phalloidin for 15 min, and nuclei were counterstained with DAPI for 7 min. Cells were imaged using laser scanning confocal microscopy (LSM-700) and a Nikon ECLIPSE Ti inverted fluorescence microscope. Thereafter, the images were analyzed using CellProfiler™ cell image analysis software (https://cellprofiler.org/). Its parameters were optimized for analysis and estimating the nuclear and cell size parameters. For cell membrane protein expression, the cells were fixed and permeabilized, as described above. Cells were incubated with fluorophore-tagged antibodies (Thermo Fisher) for cell membrane proteins, including occludin, ZO-1, claudin, and P-cadherin. After the counterstaining with DAPI, the stained cells on inserts were mounted on glass slides and observed on a confocal laser scanning microscope. The expression of each cell membrane protein was analyzed by ImageJ software. For quantification of the protein expression, the mean grey value from 30 independent cells was analyzed (*n* = 30) and compared with cells cultured on TCPS.

### 2.6 Wound healing assay

For the *in vitro* wound-healing assay, the cells cultured on the NCCI or TCPS were suspended with trypsin/EDTA solution. 1.5 × 10^5^ cells per well were re-seeded in a 24 well plate. After 24 h, the wound was created by scratching the well surface with a 200 μL pipette tip. The medium was replaced, and the initial wound area image was captured using a bright field microscope. Thereafter, the images of the covered wound were captured every 6 h. The total wound area was calculated with ImageJ software for three images at every time point. Wound healing was calculated as a fraction of the wound area with respect to wound area at *t* = 0 h.

### 2.7 Biomechanical characterization of cells

A Bio-AFM (Park System, South Korea, AFM – NX10 model and Park Systems XE-BIO AFM) combined with an inverted optical microscope and an *X*–*Y* flat scanner (100 μm × 100 μm) was used in contact mode to assess the stiffness of the tissue-like cell sheet generated after culture of HaCaT cells for 2 days on the TCPS or NCCI. The cell culture medium was drained and replaced with PBS to minimize dehydration during the measurements. The AFM was calibrated before any measurement in a fluidic environment using a spherical colloidal probe (SiO_2_ sphere of 5.2 μm diameter (AppNano USA) to minimize damage to the cells).^35^ The samples were scanned to identify the position of the cells, and nanoindentation was performed to determine the stiffness. The indentation depth was set to a maximum of ≈40% of the average height obtained after AFM imaging. The force–displacement data were recorded for the nanoindentation test performed at different locations (cytoplasm, nucleus, and cell–cell junction) on HaCaT cells. Manufacturer-provided XEP and XEI software were used to operate, analyze and store the data. Furthermore, the data obtained were analyzed through an algorithm in MATLAB 7.4 (MathWorks, Inc.) to estimate the contact point, and thereafter, the data were modeled for the estimation of different mechanical parameters.

### 2.8 Statistical analysis

All the data are presented in the format of average ± standard deviation. A paired *t*-test was applied to determine statistically significant differences (*p*-value < 0.05).

## 3. Results and discussion

### 3.1 Design, fabrication, and characterization of the nanofibrous cell culture insert

**Fig. 1:**
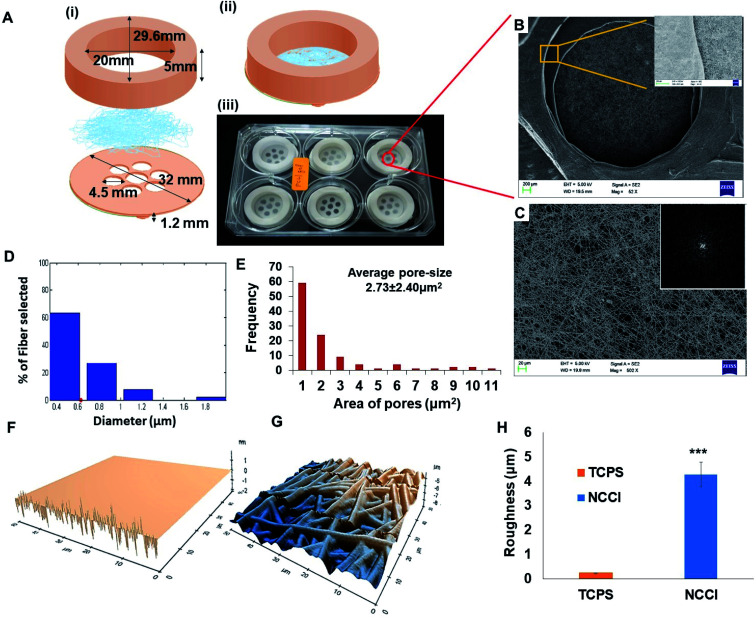
(A) (i and ii) Schematic of (i) the exploded design of the nanofibrous cell culture insert (NCCI) components and (ii) isometric view of the assembled NCCI; (iii) photograph of the NCCI placed in a six-well plate; (B) SEM of an eye of an NCCI; the inset shows the magnified view of the edge of the eye; (C) SEM of nanofibers in the eye region and the inset image is the FFT of the SEM at the edge of an eye. Graphs showing the frequency distribution of (D) diameter and (E) pore-size in the nanofibrous membrane of the NCCI. 3D AFM image of the surface of (F) TCPS and (G) the nanofibrous matrix in the NCCI. Graph showing (H) the comparison of the roughness of TCPS with the NCCI. Scale bar (B) = 200 μm and (C) = 20 μm and inset image scale bar = 5 μm; statistically significant difference in the NCCI group compared with the TCPS group (control), *** indicates *p*-value < 0.001.

Toward the development of an *in vitro* tissue model of the skin and other epithelial cells, this study focused on developing a designer microdevice, hereafter referred to as the nanofiber-based cell culture insert (NCCI) (Fig. 1A). The NCCI was designed to serve as a transwell for regenerating the skin tissue *in vitro* that could serve as a disease model and a platform for screening molecules, such as drugs, cosmetics, *etc.* The device was fabricated by integrating microfabrication techniques, including electrospinning and 3D printing (Fig. S1 and S2†). The structural part of the NCCI was prepared by fused filament fabrication (FFF) of poly(lactic acid) (PLA), while the functional part consisted of a nanofibrous PCL/Gel membrane. Fig. 1A(iii) presents the NCCI fabricated for a 6-well plate culture that was used here. The base of the device containing the nanofiber membrane was designed to lie 1.2 mm above the underlying TCPS well plate surface and support the cells cultured in the insert, as shown in Fig. 1A(i). There are seven “eye” regions, each of 4.5 mm diameter, in the NCCI where the nanofibrous membrane is not supported by the underlying PLA substrate and serves as a freely hanging membranous structure. A closer investigation of the “eye” regions of the NCCI revealed the presence of a random, non-woven 3D nanofibrous PCL/Gel matrix (Fig. 1B). The inset of Fig. 1B suggests that the nanofibers were evenly distributed. The fast Fourier transform (FFT) of the image in Fig. 1C presented in the inset confirmed that the orientation of the nanofibers was highly random. The diameter of the nanofibers was 622 ± 31 nm (mean ± S.D.), with the full distribution presented in Fig. 1D. The pore-size distribution estimated from analysis of the SEM images of the nanofibers is compiled in Fig. 1E. The average pore size in the membrane (2.7 ± 2.3 μm) was markedly smaller than the size of the keratinocytes studied here. The nanofibrous membrane with micro-sized pores provided multi-scale topography ranging from micro- to nano-scale to the cells. AFM analysis (Fig. 1F and G) revealed that the roughness (RMSD value) of the membrane is 17 fold higher than that of TCPS (Fig. 1H).

Unlike the commercially available cell culture inserts, which contain a flat porous membrane, herein, we have integrated a free-hanging nanofibrous membrane for supporting the cells in the designer insert. In previous studies, nanofibrous membranes of PCL/Gel were used to culture cells for a discriminative understanding of cell–material interactions and biophysical changes of skin cells. These composite nanofibrous membranes have been reported to promote proliferation, spreading, and integration with the underlying nanofibrous membrane of adult human skin-derived precursor cells (hSKPs) with the remodeling of the ECM *in vitro* and *in vivo*.^36^ The nanofibrous membrane was integrated with a 3D printed device to form the NCCI. The use of FFF for fabrication affords a user-defined geometry of the devices.^37^ The device can be easily adapted for various sizes of wells. Additional changes in the design to optionally introduce a groove to place electrodes for measuring TEER can be easily implemented through 3D printing. The “eye” region of the NCCI has small and tortuous pores in the nanofibrous membrane, which enables the passage of the liquid culture medium while limiting the passage of the HaCaT cells across the nanofibrous membrane. In addition, FEM analysis of the representative volume element (RVE) of the “eye” region of the free-hanging membrane suggests the presence of pre-stressed conditions with ∼0.5 μm of maximum membrane displacement (Fig. S3 and S4†). Thus, HaCaT cells may sense a mechanical stimulus from the “eye” region of the NCCI in a manner reported by Panzetta *et al.* where they suggested that mechanosensing through differential strain energy in the substrate leads to expression of the stiffer cytoskeleton in murine fibroblasts and preosteoblasts.^38^ Furthermore, the stiffness of the biocomposite nanofibrous membrane (0.12 kPa) is several orders less than that of TCPS. The altered physical microenvironment offered cues for cellular spreading and promoting differentiation towards osteogenic lineage.^39^ In addition, the binding of cell integrins with RGD of randomly distributed nanoislands of gelatin in the biocomposite nanofiber membrane can potentially occur in all directions but in a limited number. Consequently, the spreading of HaCaT cells is restricted, thereby imparting a more spherical morphology.^40^

Meka *et al.* reported that the topography of the nanofibrous membrane plays a vital role in the cellular adhesion, migration, and proliferation of stem cells.^18^ Increased roughness of the nanofibrous mats compared to the smooth TCPS surfaces (Fig. 1H) can influence the cellular mechanoresponsive behavior. Moreover, several varieties of cells are known to be sensitive to the stiffness of the underlying substrate.^21^ Thus, the nanofibrous substrate of the NCCI offers a unique combination of low stiffness and high roughness to the supporting cells. Furthermore, the NCCI serves as a barrier membrane where the media can freely diffuse across the membrane initially.^41,42^ Thus, it was envisaged that the unique design attributes of the NCCI could better recapitulate the functions of the keratinocytes and other epithelial cells.

### 3.2 Changes in the cell and nuclear morphologies on the NCCIs

**Fig. 2:**
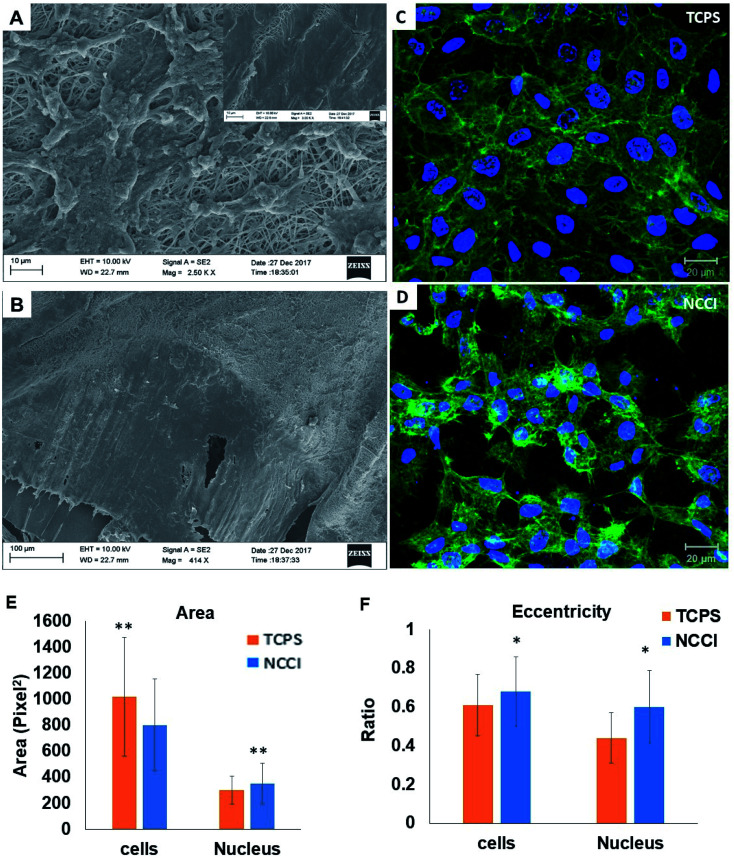
(A and B) SEM images of (A) HaCaT cells grown on nanofibers of the NCCI and (B) cross-section of the insert showing a monolayer of confluent HaCaT cells; (C and D) fluorescence micrograph of HaCaT cells on (C) TCPS and (D) the NCCI showing the nucleus (blue) stained with DAPI and actin (green) stained with Alexa Fluor 546 phalloidin; (E and F) graph showing the variation of (E) area and (F) eccentricity of cells and nuclei of HaCaT cells grown on the TCPS and NCCI; scale bar (A and B) = 100 μm, inset image = 10 μm and (C and D) = 20 μm; * indicates *p*-value < 0.05 and ** indicates *p*-value < 0.01, *n* = 50.

**Fig. 3:**
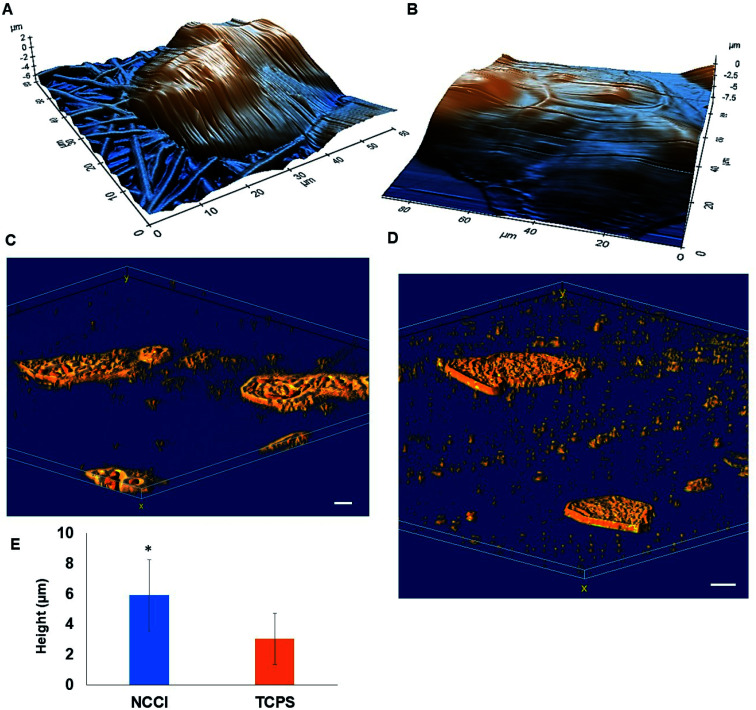
(A and B) 3D topographical AFM images of HaCaT cells grown on (A) the NCCI and (B) TCPS; 3D z-stacked confocal images of HaCaT cells grown on (C) the NCCI and (D) TCPS. (E) Graph showing the height of the HaCaT cells grown on the NCCI and TCPS; scale bar (A and B) = 10 μm and (C and D) = 20 μm; statistically significant difference in the NCCI group compared with the TCPS group (control), * indicates *p*-value < 0.001, *n* = 10.

HaCaT cells are human skin-derived keratinocytes that are widely used as a model for the study of homeostasis and pathophysiology of the skin epidermis. Scanning electron micrographs reveal that the cells adhered and proliferated well on the membrane in the NCCI to confluence within 3 days, as seen in top-view (Fig. 2A) and cross-sectional images (Fig. 2B). Fluorescence micrographs of the F-actin and nuclei of the cells on the TCPS and NCCI (Fig. 2C and D) confirmed the formation of the confluent cell sheet. These images were processed to quantify the area, eccentricity, and compactness of the cells and their nucleus. The area of the cells grown on TCPS is higher as compared to that on the NCCI (Fig. 2E), while the area of the nucleus is lower on TCPS (Fig. 2E). Compactness, which measures the spherical nature of the cells, is higher for the cells on the NCCI than on TCPS (Fig. S5A†). The eccentricity of the cells and nuclei on the NCCI is closer to 1.0 as compared to cells on TCPS (Fig. 2F), confirming the more equiaxial shapes of the cells and nuclei on the NCCI and corroborating the compactness data. The inter-nuclear distances among the cluster of cells are lower on the NCCI than on TCPS (Fig. S5B†). Taken together, the HaCaT cells are more compact and closely spaced into cell sheets when grown on the NCCI. A Bio-AFM was used to probe the live cells on the nanofibers of the NCCI and cells on TCPS to yield 3D images of the cells, as shown in Fig. 3A and B. The z-stack composite images of the cells grown on the NCCI and TCPS were also obtained using the confocal microscope (Fig. 3C and D). AFM analysis reveals a three-fold increase in the height of cells on the NCCI compared to cells on TCPS (Fig. 3E), further confirming a more compact morphology of the cells on the NCCI.

The higher projected area of cells grown on TCPS is due to the enhanced spreading of cells in the 2D culture owing to the planar morphology of the substrate and the formation of more focal adhesions.^40^ It is reported that increased substrate stiffness and lower roughness promoted enhanced spreading of fibroblasts and human mesenchymal stem cells (hMSCs), respectively.^21,43^ However, the fibrous architecture of the NCCI offers higher roughness and lower stiffness than TCPS to the HaCaT cells. Moreover, the porous nanofibers offer fewer cell-binding sites for the formation of focal adhesions and minimal strain energy leading to reduced cell spreading.^44^ The increased size of the nucleus and higher nuclear-to-cytoplasm area ratio of the cells on the NCCI than the cell on TCPS underscores the role of the substrate properties in modulating the cellular behavior.^18^ It is well recognized that cellular mechano-transduction induces changes in the nuclear morphology (size) with altered mitotic activity, gene expression, migration, plasticity, and cytoskeletal contractility resulting from alterations in the physical microenvironment.^45^ The increased compactness and eccentricity of the cells on the NCCI than on TCPS surfaces observed here corroborate earlier studies. Beijer *et al.* demonstrated that oriented microtopographies increased eccentricity in hMSCs, resulting in reduced cellular metabolism and retarded cell cycle progression, accounting for low cell proliferation.^46^ In our earlier work, we observed that primary cardiomyocytes on aligned microtopographies exhibited a native tissue-like phenotype than cells on TCPS, and these differences were associated with changes in nuclear morphology.^47^ The heights of the cells from the confocal images on the NCCI (height ≈ 15.56 μm) and TCPS (height ≈ 8.99 μm) were found comparable but not identical. Such difference arises due to the difference in the techniques for obtaining the 3D height of the cells associated with individual methods. However, we can observe a trend in the difference in heights. The confocal and AFM images reveal the 3D morphology of HaCaT cells on the NCCI in contrast to the flattened morphology on TCPS, further corroborating the compactness and roundness calculated from analysis of the fluorescence images (Fig. 2C and D).

### 3.3 Altered cellular nanomechanics of the keratinocytes on the NCCIs

**Fig. 4:**
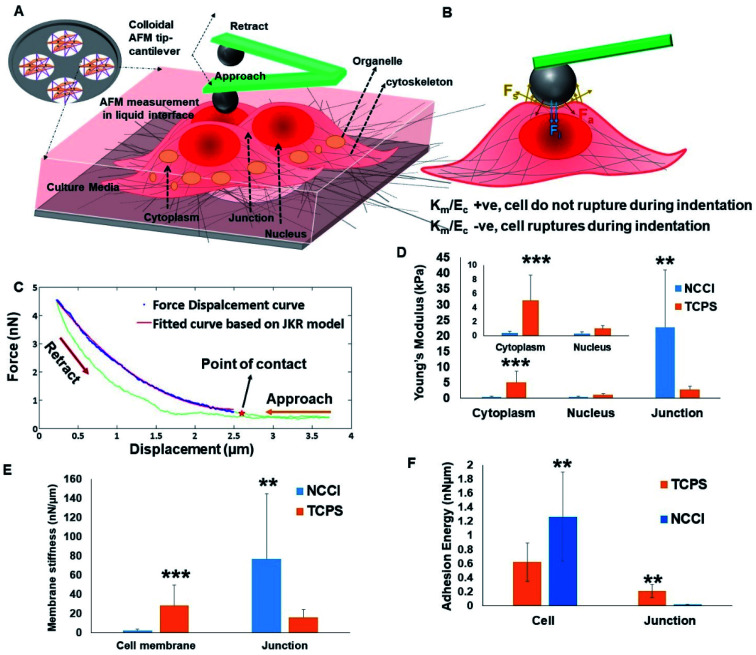
(A) Schematic of Bio-AFM imaging and nanoindentation of live HaCaT cells grown on the NCCI; (B) schematic showing the nature of forces acting on the cells during the process of nanoindentation, including (1) a colloidal probe applying a compressive force (*F*_i_) on a cell of bulk modulus *E*_c_, (2) membrane stretches by Δ*x* due to the compressive force exerted by the probe, (3) resistive force offered by a membrane of stiffness *K*_m_ due to stretching and surface energy minimization (*F*_s_), (4) force exerted on the probe due to adherence with the stretched part of the membrane (*F*_a_); (C) graph showing the force–displacement curve during the nanoindentation test using a Bio-AFM; (D–F) graphs showing the variation of (D) the Young's modulus of the cytoplasm, nucleus and cell-to-cell junctions in HaCaT cells grown on the NCCI and TCPS, (E) membrane stiffness of HaCaT cells of the cell membrane and cell-to-cell junctions grown on the NCCI and TCPS, (F) adhesion energy released when the nanoindenter interacts with cells and the junction of HaCaT cells grown on the NCCI and TCPS; statistically significant difference in the NCCI group compared with the TCPS group (control), ** indicates a highly significant *p*-value < 0.01, *** indicates the most significant *p*-value < 0.001.

To probe possible differences in the mechanical properties of the developing cell sheets on the TCPS and NCCI, a Bio-AFM was used to probe the cytoplasm, the nucleus, and the cell-to-cell junctions of the cells (Fig. 4A and B). The force–displacement plots are compiled in Fig. 4C. The force experienced by the AFM tip while indenting the HaCaT cells on both the substrates exhibits a parabolic relationship with displacement, suggesting non-linear elastic deformation (Fig. S6†). Furthermore, a monotonic non-linear increase in the resistance force is offered by cells during the approach of the AFM tip on indenting the cytoplasm or nucleus on either substrate (Fig. 4C). However, during the retraction of the tip, the curves do not follow the approach curve indicating strong adhesion between the tip and the cells (Fig. 4C). Therefore, the force–displacement curve was modeled following the JKR theory of contact mechanics of soft tissues. The Young's modulus of different cellular parts, namely, the cytoplasm, nucleus, and cell-to-cell junction of cells grown on the TCPS and NCCI, was estimated from the JKR model and is presented in Fig. 4D. The comparative analysis of surface stiffness of the tissue components is shown in Fig. 4E after estimation from JKR theory. It was observed that the ratio of stiffness of the membrane to bulk modulus is 0.0314 in the cells grown on TCPS compared to 1.37 for the cells on the NCCI. Fig. 4F shows that the adhesion energy released during bonding of the cytoplasm and nucleus with the nanoindenter is relatively higher on the NCCI than for cells grown on TCPS.

The force–displacement behavior of the developing tissue shows the characteristics of indentation of a soft, sticky material by a stiff indenter.^48^ The JKR model equation adapted from Style *et al.* fitted well with the force–displacement curve obtained from the AFM analysis.^49^ Thus, JKR theory is applied to estimate the mechanical behavior of the indented live cells. It is seen that the Young's modulus is significantly lower for the cytoplasm and nucleus of the cells grown on the NCCI than those on TCPS (Fig. 4D). The NCCI offers a compliant, porous substrate with limited cell-adhesive sites for the cells that minimize cell spreading and induce changes in the cytoskeletal organization compared to cells on TCPS with supra-physiological stiffness and a flat architecture.^50^ There are several reports in the scientific literature on changes in mechanical properties of cells in response to a difference in the physical microenvironment. Rother *et al.* reported that the MDCK-II epithelial cells grown on micro-/nano-porous substrates exhibit lower stiffness and fluidic nature than cells on the planar TCPS plates.^51^ It has also been reported that the mechano-responsive cells with less stiff nuclei tend to migrate easily and adapt to the changes in substrate stiffness through reorganization of cytoskeletal elements.^52,53^ Lower stiffness of the nuclei of the cells cultured on the NCCI than cells on TCPS can be expected to result in phenotypical differences such as slower proliferation and higher migratory potential compared to cells on TCPS.^54^ Notably, the Young's modulus of the cell-to-cell junction was higher for cells on the NCCI as compared to TCPS (Fig. 4D). The thickness of the junction is sufficiently higher (2.1 ± 0.6 μm) as compared to the indentation depth of the spherical probe, which was 100 nm. Furthermore, the stiffness of the cell-to-cell junction (Young's modulus = 30.73 Pa) obtained is markedly lower than that of the underlying nanofibrous substrate (118.1 Pa). This is possible only when the probe does not experience the stiffness of the underlying substrate and measured cell-to-cell junction stiffness. It appears that the thicker, less spread cells on the NCCI interact more intimately with adjacent cells to form mature cell junctions *via* increased expression and localization of the associated membrane proteins. These increased interactions lead to increased stiffness of the junctions when the cells are grown on the NCCI than on the flatter TCPS, forming immature and less stiff cell-to-cell junctions. It is believed that tight junctions enhance the cross-talk between cells and are key for controlling the selective transport of small molecules.^55^

The quadratic nature of the force–displacement plot results from the contribution of the cell membrane to the measured nanomechanical properties of cells. The membrane stiffness is a function of the maturity of the cell membrane and the presence of cytoskeletal elements.^56^ The increased fluidic properties of a cell are associated with a higher potential for migration.^57^ It has been reported that fluid-like behavior plays a crucial role in normal physiology such as growth and repair, lung expansion, blood filtration, muscle contraction, *etc.* At the same time, fluidic behavior may also be associated with pathophysiological states such as kidney disease, focal segmental glomerulosclerosis, cancer metastasis, *etc.*^57^ Lee *et al.* showed that as soft substrates can be easily deformed by cells, cells retain their fluidic behavior for migration and save energy on podia formation, which is the common mode of migration on stiff substrates.^58^

A parameter that affects the mechanics of cells in tissues when probed with a nanoindenter is the adhesion energy. The adhesion of the cell membrane to a nanoindenter is due to the fluidic nature of the cells, their chemical interactions, and van der Waal's forces.^59^ The adhesion energy was estimated for the cells on the TCPS and NCCI by fitting the AFM data to the JKR model. The higher adhesion energy of cells grown on the NCCI indicates that cells are of a fluidic nature and form a monolayer compared to the cell layer on TCPS. The higher adhesive energy might be due to the interaction of cells with the neighboring cells and the underlying substrate, indirectly suggesting a better monolayer of tissue formation. Similar findings were reported by Sancho *et al.* who observed higher adhesion force experienced by the AFM tip on probing a monolayer of human endothelial cells from the umbilical artery compared to a single cell.^60^ However, it was observed that the adhesion energy liberated at a cell-to-cell junction on a TCPS is higher than that at the NCCI junction point. The formation of more mature cell-to-cell junctions on the NCCI may have caused local stiffening such that the cells on TCPS were more fluidic at the edges than the cells on the NCCI, leading to the observed differences in the adhesion energy.

### 3.4 *In vitro* performance of keratinocytes

**Fig. 5:**
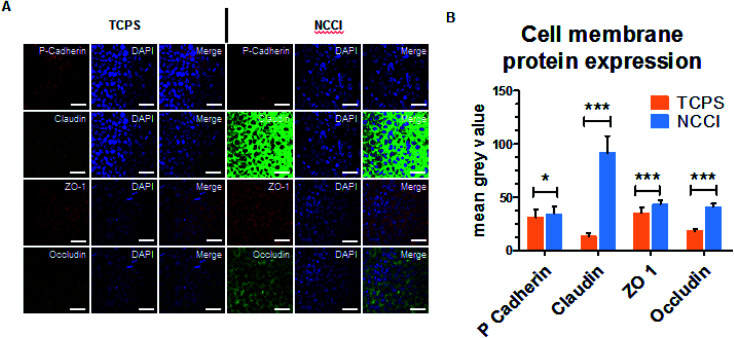
(A) The expression and localization of cell membrane proteins using anti-occludin goat, anti-claudin goat, anti-ZO-1 mouse, and anti-p-cadherin mouse primary antibodies and secondary antibody rabbit anti-goat FITC and Alexa Fluor® 594 goat anti-rabbit on HaCaT cells grown on the TCPS and NCCI (scale bar = 50 μm); (B) graph of the mean grey value for quantification of each protein in individual cells on the TCPS and NCCI determined by analyzing the z-stack confocal images in ImageJ software (*n* = 30 cells for each protein); statistically significant difference in the NCCI group compared with the TCPS group (control), wherein * indicates a significant difference at *p* < 0.5 and *** indicates a highly significant difference at *p* < 0.001.

**Fig. 6:**
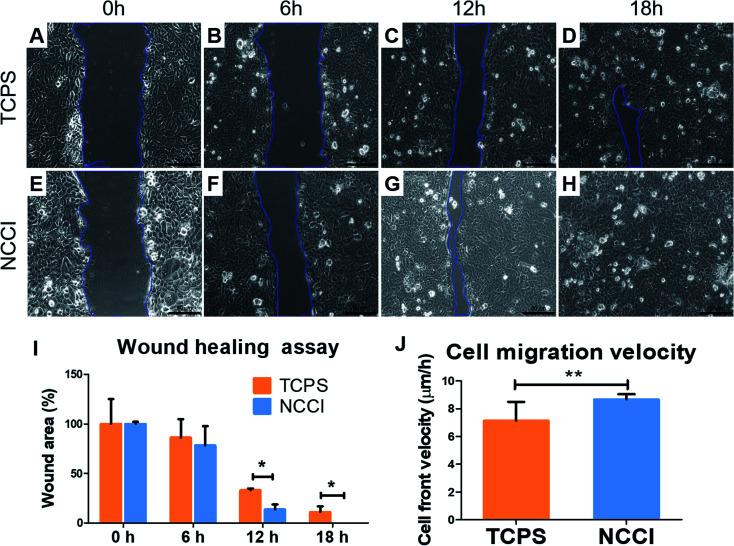
Migration of HaCaT cells analyzed by the wound healing assay; the assay was performed in 24 well plates with HaCaT cells either pre-grown on the 2D TCPS control or NCCI. (A–D) The wound area for HaCaT cells from TCPS at the respective time points; (E–H) the wound area of the NCCI grown HaCaT cell at the respective time points. Scale bar 80 μm. (I) Graphs showing the percentage wound area in these images, and (J) the migration velocity of cells during the wound healing process in the TCPS and NCCI; * and ** indicate a significant difference at *p* < 0.05 and *p* < 0.01, respectively.

To assess whether the differences in the nanomechanical properties of the cells on the NCCI and TCPS are associated with any phenotypical changes, we semi-quantitatively assessed the expressions of cell junction proteins, including P-cadherin, claudin-1, cadherin, and ZO-1 by immunostaining. The fluorescence images (captured at the same settings of the microscope and camera for a given protein) show a marked elevation in the expressions of all these proteins on the NCCI than TCPS (Fig. 5). Specifically, ImageJ software used for quantification of the fluorescence intensity revealed that claudin-1 and occludin expressions were 6.8 and 2.3 fold, respectively, amplified on the NCCI. Herein, we observed the formation of dense cellular organization with enhanced intercellular adhesions on the NCCI. The elevated expression of the junction proteins suggests the formation of tighter cell–cell junctions that imparted greater stiffness at the cell-to-cell junctions, as revealed by AFM measurements described above. These observations are in good agreement with the non-canonical functions of claudin expression.^61^ To further assess the functional changes associated with the alterations in the cellular nanomechanics in the NCCI, we performed a scratch assay. The cells from the NCCI showed a higher ability to migrate *in vitro* than cells on TCPS (Fig. 6). The collective cell migration rate on the NCCI was considerably higher and exhibited significantly faster wound closure, and the wound was completely covered within 18 h, unlike cells cultured in TCPS. This enhanced collective migration of cells likely results from enhanced communication among cells, as evidenced through the higher expression of cell junction proteins in the NCCI.

The nanofibrous substrate of the NCCI provides a conducive platform to investigate the cellular behavior of keratinocytes that better recapitulates the biomechanical and functional phenotype of the cells than when cultured on TCPS substrates. Cell adhesion proteins, specifically tight junction proteins, are transmembrane proteins involved in intracellular signaling molecules.^62^ These junction proteins preserve tensional homeostasis and participate in paracrine signaling.^63^ Knockdown of these proteins impedes intracellular signaling underlying various biological phenomena. For example, knockdown of claudin-1 downregulates p-AKT and impairs the migration of skin keratinocytes.^64^ Claudins also constitute paracellular pores for selective ion transport that govern inflammatory response and tissue remodeling.^65^ Thus, the expression and functioning of cell adhesion molecules are critical to the success of the skin tissue model. The ability of the NCCI as a platform to recapitulate cellular organization and formation of cell–cell junctions underscores the utility of this device for realizing the long-term goal of engineering *in vitro* mimics of human epithelial tissues with skin keratinocytes as an example.

It is reported that cells maintain tight intercellular adhesions and migrate collectively as a monolayer for healing wounds.^66,67^ Notably, these processes of collective cell migration are conserved in several wound healing processes, including those of the skin, cornea, digestive epithelium, and endothelium.^66,68,69^ The higher cell proliferation and better cell–cell communication could be the governing factor for faster wound healing. The PCL-based material is reported as the better substrate to provide hydrophilic properties for adhesion and activate proliferative markers.^70^ Moreover, in the NCCI developed herein, we observed that the keratinocytes express higher levels of key cell membrane proteins involved in cell–cell adhesion and exhibit accelerated wound healing compared to cells on TCPS. The collective effect of higher cell proliferative state and improvised cell–cell communication resulted in faster wound closure by keratinocyte cells.^71^ Thus, the NCCI was able to stimulate the cells to augment tissue formation even in the absence of biomolecules through biophysical stimuli such as lower and gradient stiffness and higher roughness than in the case of TCPS. Taken together, the NCCI designed here is demonstrated to be a promising platform for engineering a skin tissue model *in vitro* compared to conventional culture techniques.

## 4. Conclusion

A novel NCCI was designed and fabricated to enable cell culture over a free-hanging nanofibrous membrane that can mimic the architecture of the ECM. Keratinocytes cultured in the NCCI exhibited a compact 3D cellular morphology, higher expression of cell-to-cell junction proteins, and improved cellular migration ability compared to cells in the 2D TCPS culture. The biophysical attributes of the cells measured by nanomechanical characterization of the live cells reveal softer cells and nuclei with stiffer cell-to-cell junctions on the NCCI than on TCPS. These biomechanical and biochemical observations are hallmarks of native skin tissue generation and demonstrate the ability of the NCCI to serve as a better platform for epithelial tissue formation *in vitro*.

## Conflicts of interest

The authors have no conflicts to declare.

## Supplementary Material

NA-003-D1NA00280E-s001
