# Supplementary material for: A designer cell culture insert with a nanofibrous membrane toward engineering an epithelial tissue model validated by cellular nanomechanics
Source: Nanoscale Adv. 2021 Jul 5;3(16):4714–25. doi: 10.1039/d1na00280e (PMC9419865; doi:10.1039/d1na00280e)
Supplement: NA-003-D1NA00280E-s001 [file NA-003-D1NA00280E-s001.pdf]

Electronic Supplementary Material (ESI) for Nanoscale Advances.
This journal is © The Royal Society of Chemistry 2021

Supplementary Information

Designer Cell Culture Insert with Nanofibrous Membrane toward Engineering an Epithelial Tissue Model Validated by Cellular Nanomechanics

Prasoon Kumar[1,3], **Dhaval Kedaria**[1], **Chinmaya Mahapatra**[1,4], **M. Monisha**[2], **Kaushik Chatterjee**[1,2]

[1]Department of Materials Engineering and [2]Centre for BioSystems Science and Engineering, Indian Institute of Science, C.V. Raman Avenue, Bangalore, India 560012

[3]Department of Biotechnology and Medical Engineering, National Institute of Technology, Rourkela, India 769008

[4]School of Chemical Engineering and Biomedical Institute for Convergence at SKKU (BICS), Sungkyunkwan University, Suwon 16419, Republic of Korea

1. Design and Fabrication of NCCI

We designed and fabricated nanofiber-based cell culture inserts by combining two scalable micro-/nano- technologies, electrospinning and 3D printing. The structural parts of cell culture inserts were fabricated by a 3D printer consisting of two components prepared from poly(lactic acid) (PLA). The diameter and the thickness of the base component (disc) are 64 mm and 0.6 mm, respectively. There are four legs with 0.5 mm in height and 1 mm in diameter connected to the base component. They serve as supports to lift the inserts above the base of the culture plates. The disc has holes of 4.5mm in diameter (Figure S1B). These structural parts of inserts can be fabricated into any dimension, depending on the resolution of the 3D printer. Thereafter, the supports of NCCI were dipped in electrolyte (0.01M KCl) solution and placed over a Whatman filter paper soiled with the electrolyte, as shown in Figure S1A. This arrangement created uniform conducting regions on the NCCI during the electrospinning process. Consequently, fibers were uniformly deposited on the eye part of the base component of the NCCI, as shown in Figure S1D, unlike the setup where nanofibers

were deposited without the use of electrolyte (Figure S1C). The micropore size distribution of the conventional commercial inserts (Figure S1E) was observed to be nearly uniform, unlike for NCCI. It was observed that nanofibers were radially aligned due to uneven stretching of nanofibers resulting from the differential spatial variation of the electric field strength near the edge of an eye of the NCCI when electrospinning was performed without the electrolyte. However, the use of the electrolyte minimizes the spatial variation in the electric field over the entire region of the eye. Thus, the uniform increase in the conductivity of the PLA substrate with the use of electrolyte results in a uniform fiber deposition, which is evident from the SEM images[1]. Due to the use of electrolyte, there might have been an increased humidity inside the electrospinning chamber. The conductivity of humid air is greater than dry air. The humid air also facilitates the uniform deposition of nanofibers. Moreover, PCL/gelatin nanofibers being hydrophilic in nature, their nanofiber mat remained in wet condition due to capillary wicking of electrolyte and supported the layer-by-layer deposition without any decrease in conductivity of the substrate. After the uniform deposition of nanofibers of PCL/gelatin on the entire surface of the base component, they were bonded to its boundary component with partially cured PDMS glue. The PDMS was used as glue due to its inert, non-toxic nature during cell culture. Further, the PDMS glue resulted in leak-proof bonding and afforded characterization of the cells by dismantling the components of the NCCI. We have systematically varied the parameters of 3D printing, electrospinning, and assembly of the components for optimal fabrication that is simple, scalable, and inexpensive. Furthermore, we observed that ethylene oxide sterilization of the NCCIs was better than the use of ethanol.

2. FEM modeling and simulation for optimization of nanofiber deposition

The electrospinning of nanofibers on a substrate is dependent on its conductivity, and the pattern of deposition is primarily governed by the substrate chosen for deposition. The metallic substrates have been used conventionally to deposit the fibers, and thereafter, the fibers are peeled off for different applications. However, it is challenging to skillfully peel off the nanofibrous membrane and integrate it with other non-conducting substrates. Thus, it is imperative to develop strategies to deposit nanofibers on non-conducting polymeric substrates. Therefore, we have simulated the electric field and electric potential between a

needle and a substrate under a steady-state condition through a finite element method in COMSOL Multiphysics 4.2[2]. We defined the two-dimensional geometry of needle and substrate with air as a dielectric medium between them (Figure S2A & S2B). The two cases have been investigated; one, where the substrate is a non-conducting polymeric material, and second, where a non-conducting polymeric substrate is covered with a conducting electrolyte layer.

Polymeric nanofibrous membranes were fabricated by the electrohydrodynamic drawing of polymers in an external electric field on a non-conducting substrate through an electrolyte-assisted electrospinning process. The nanofibers were deposited on a non-conducting substrate having alternate regions of conductivity. It can be observed that the strength of electric field distribution (different length of the red arrow) in the conductive and non-conductive regions were different, as shown in Figure S2C & S2D. It is due to the high dielectric constant of the non-conducting substrate that the electric field was concentrated to a greater extent in the exposed conducting part of the substrate as compared to non-conducting regions, which results in a differential electric field formation on a substrate. This method can be utilized for manipulating or patterning the electric field on a substrate in an electric field, thereby, can be used for nanofibers or nanoparticles patterning during the electrospinning process. However, the use of a thin electrolyte layer over the non-conducting substrate leads to a contiguous electric field on the substrate. This can be clearly observed by the uniform length of the red arrow near the substrate surface, irrespective of the conducting and non-conducting regions. The spatial distribution of electric field strength near the surface of the substrate (0.5 mm from the substrate) shows a sinusoidal and uniform pattern of the electric field on the non-conducting substrate without and with electrolyte, respectively (Figure S2E and S2F). Moreover, the strength of the electric field due to the use of the electrolyte was higher as compared to one without the electrolyte. The equation describing the fluctuation of the electric field near the surface of the substrate without an electrolyte is given by

$$E(x) = Eo + E_A sin\{\frac{\pi(x - xc)}{\omega}\} \quad \text{(eq. 1)}$$

Where Eo is the basic electric field.

It can be simulated that a charged particle (i.e., charged nanofiber/nanoparticle) will follow different velocities at different regions of the non-conducting substrate when electrospinning without an electrolyte (Figure S2F). As a result, there may be local stretching of nanofibers due to different velocity of an infinitesimal section of the nanofibers. This might result in an alignment of nanofibers near the periphery of the conducting and non-conducting segment (Figure S1C & S1D). The velocity of the charged particles can be given by the equation below

$$V^2(x) = Vo - [Va\omega cos\left\{\frac{\pi(x - xc)}{\omega}\right\}]/\pi \quad \text{(eq. 2)}$$

$$V_o = 2qEo/m \quad V_a = 2qE_A/m$$

It can be observed that the use of a transient electrolyte during electrospinning result in the uniform velocity of infinitesimal charged regions of the nanofibers, resulting in a uniform deposition of nanofibers/nanoparticles over the substrate. After the evaporation of the electrolyte, uniform deposition of nanofibers was obtained (Figure S1C & S1D). Thus, the use of transient electrolyte and the ability to tailor the conducting and non-conducting regions of the polymeric substrate through patterning hydrophilicity can result in the deposition of nanofibers/nanoparticles during electrospinning/electrospraying.

3. FEM modeling and simulation of RVEs in NCCI

The CAD model of the free-hanging microporous membrane present in the commercial cell culture inserts, as shown in Figure S3. We have developed the NCCI with "eye" regions where the nanofibrous membrane exists as free-hanging structures. As a hanging membrane either in NCCI or commercial inserts, the membrane is always in pre-stressed condition before it is used for seeding cells. Therefore, we have used finite element modeling (FEM) to evaluate the stress distribution, available strain energy, and degree of displacement of the membrane under its own weight. We simulated the stress distribution on the representative volume element (RVE) of the hanging membrane of the commercial insert and nanofibrous membrane of NCCI under a steady-state condition using structural

mechanics module in COMSOL Multiphysics 4.2[3]. Under the constraint boundary and body loading condition, boundary conditions are employed during the FEM simulation. It was observed that strain energy density is comparatively higher in NCCI as compared to the commercial inserts (Figures S3 & S4). Apart from strain energy density, the membrane in NCCI experiences higher deformation leading to a sagging structure as compared to the commercial insert substrate (Figure S4).

4. Biomechanical theory of Nanoindentation

The empirical equation obtained from the force displacement data modeling (Figure S6) has a close resemblance with the JKR theory of contact mechanics[4]. The theory suggests that there are three components of the total energy generated by an indenter while indenting a very soft, sticky material like cells. The first component of the total energy can be given by Hertz's theory. It suggests that the elastic energy required to create an indentation depth d by a spherical indenter of a radius of curvature R is given by equation 3.

$$E_{hz} = \frac{cER^{0.5}d^{2.5}}{(1 - v^2)} \qquad \text{(eq. 3)}$$

While indenting the soft, sticky material, there is a possibility of the indenter stretching the elastic cell membrane by an indenter to create a new surface area, and the cell membrane may wrap/stick around the indenter. Therefore, an energy penalty equal to the surface tension times the additional increase in the surface area has to be included in the total energy count and can be given by equation 4

$$E_{st} = \pi\gamma_{sv}d^2 \qquad \text{(eq. 4)}$$

Finally, there is a release of energy during the indentation process that comes from the adhesion of an indenting particle to the stretched substrate. This process drives the indentation process, and the energy liberated is given by W times the adhered area (equation 5)

$$E_{ad} = 2\pi WRd \qquad \text{(eq. 5)}$$

Therefore, total energy (U) can be given by equation 6

$$U = E_{hz} + E_{st} + E_{ad} \quad \text{(eq. 6)}$$

Further substituting and minimizing the total energy, the indenting force (F) and displacement (d) can be given by equation 7

$$F = \frac{2.5cER^{0.5}d^{1.5}}{(1 - v^2)} + 2\pi\gamma_{sv}d - 2\pi WR \quad \text{(eq.7)}$$

References

1 W. S. Choi, G. H. Kim, J. H. Shin, G. Lim and T. An, *Nanoscale Res. Lett.*, , DOI:10.1186/s11671-017-2380-6.

2 J. I. Kim, J. Y. Kim and C. H. Park, *Sci. Rep.*, 2018, **8**, 1–13.

3 P. Kumar and R. Vasita, *J. Appl. Polym. Sci.*, 2017, **134**, 1–11.

4 R. W. Style, C. Hyland, R. Boltyanskiy, J. S. Wettlaufer and E. R. Dufresne, *Nat. Commun.*, 2013, **4**, 1–6.

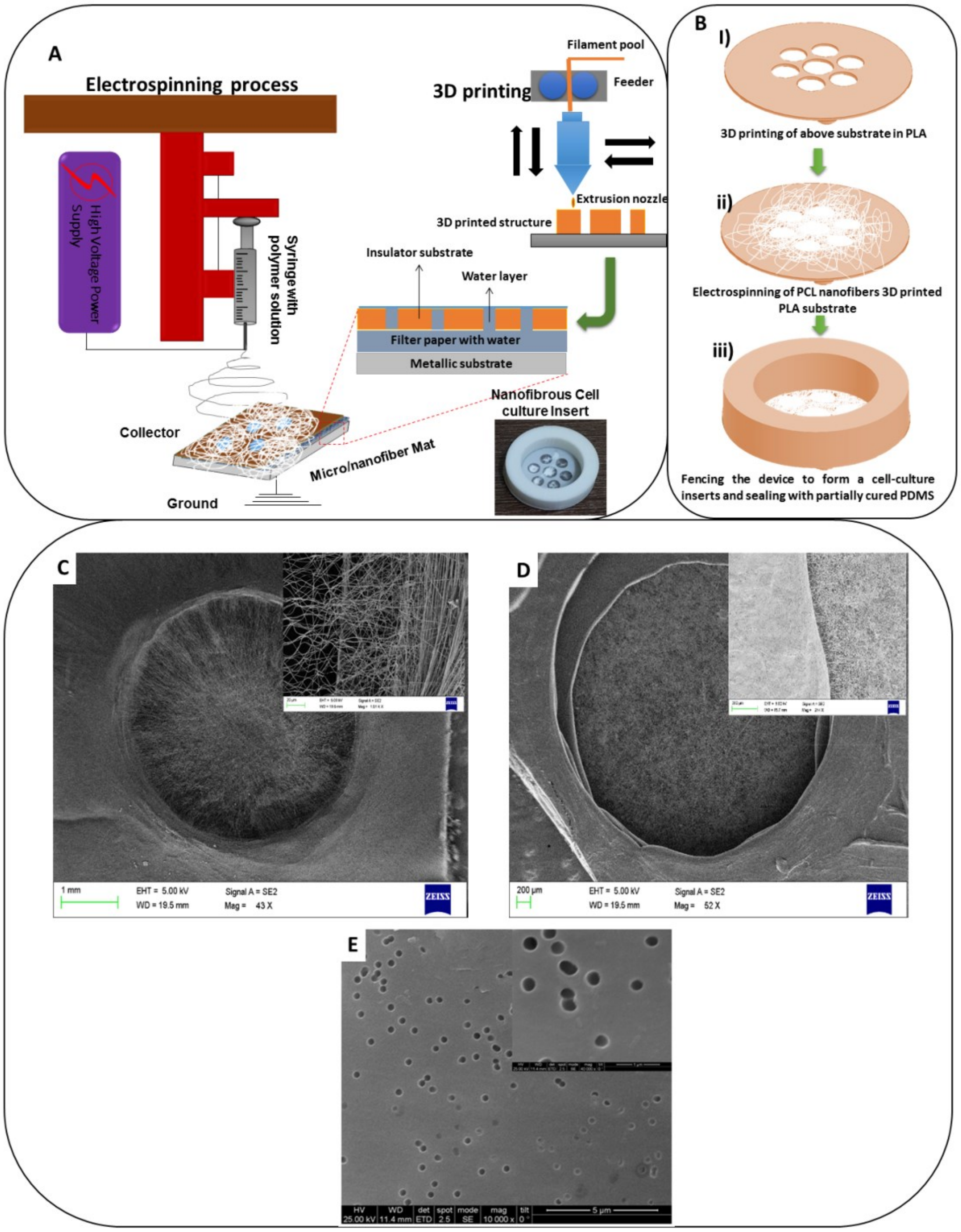

Figure S1: Schematic showing (**A)** the integration of electrospinning and 3D printer for the fabrication of the components of nanofibrous cell culture inserts (NCCI); **(B)** the sequence of integration of components of NCCI to form a functional NCCI. SEM of one of the eyes of a nanofibrous cell culture insert created; **(C)** without electrolyte assisted electrospinning (The inset image shows the magnified view of the edge of the eye); **(D)** with electrolyte assisted electrospinning and inset image is the magnified view of the edge of the eye) **E)** SEM of the microporous PC membrane of conventional commercial inserts

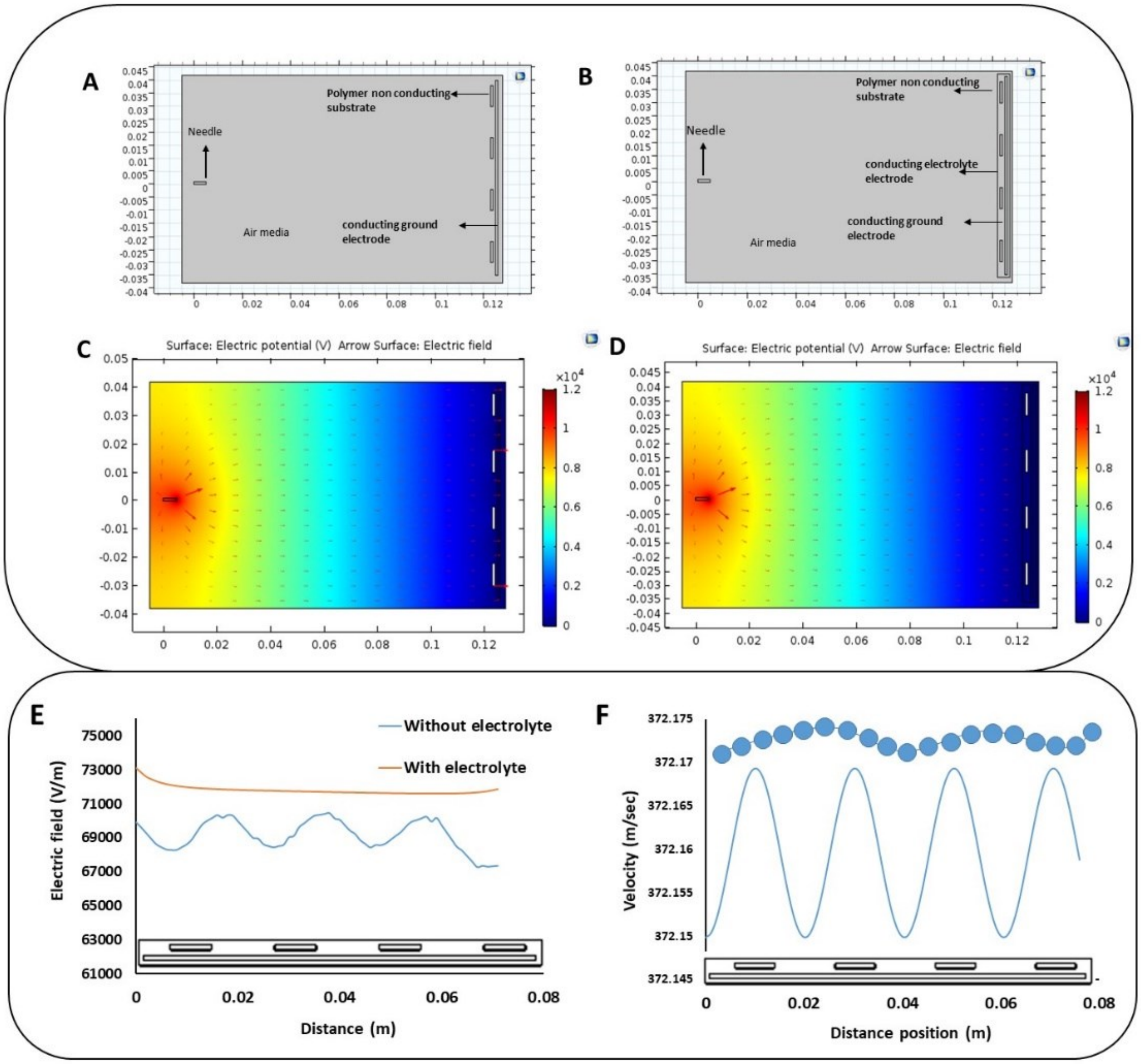

Figure S2: Schematic of 2D model simulating the electric field between a needle and grounded substrate **(A)** without an electrolyte layer and **(B)** with an electrolyte layer; A color map illustrating the distribution of electric potential and red arrow showing the direction and magnitude of electric field between needle and grounded substrate for case **(C)** without an electrolyte layer and **(D)** with an electrolyte layer. Graph showing the variation of **(E)** the magnitude of electric field and **(F)** the magnitude of velocity field over the length of 7 cm on a substrate.

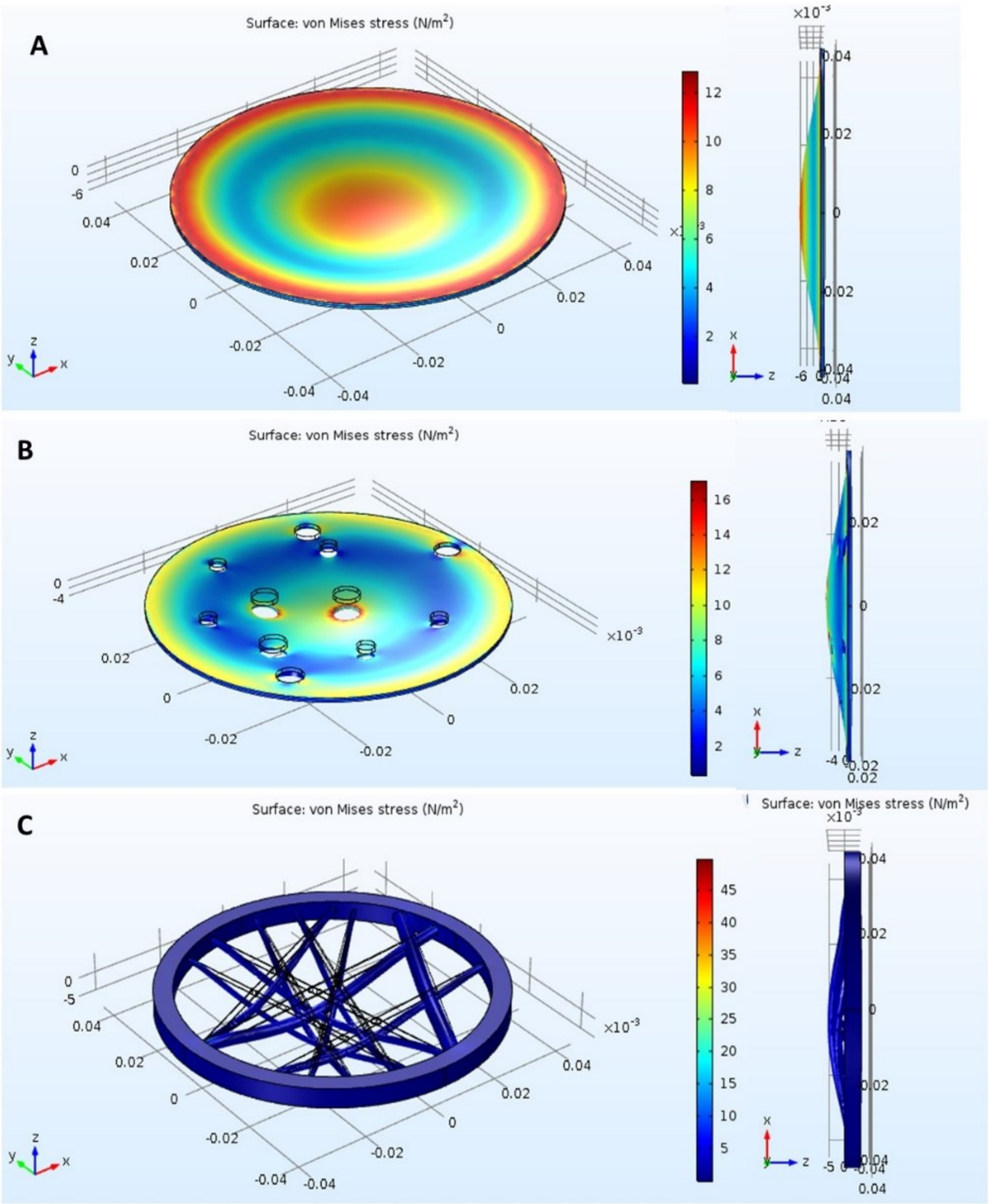

Figure S3: Stress distribution on the free-hanging substrates **(A)** polystyrene film, **(B)** polycarbonate microporous insert and **(C)** PCL nanofibrous membrane under its own weight.

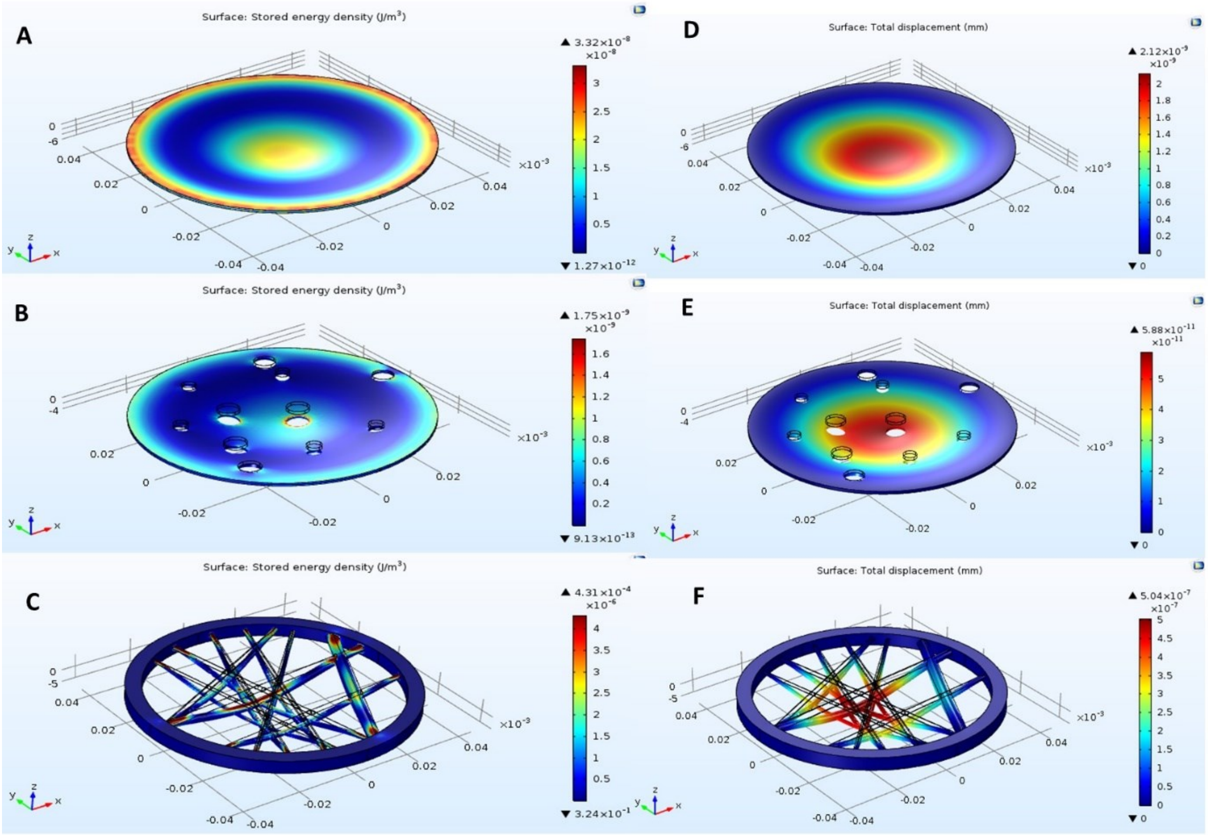

Figure S4: Strain Energy density distribution on the free-hanging substrates **(A)** polystyrene film, **(B)** polycarbonate microporous insert and **(C)** PCL nanofibrous membrane under its own weight; and total displacement of the free-handing substrates **(D)** polystyrene film **(E)** polycarbonate microporous insert and **(F)** PCL nanofibrous membrane under its own weight

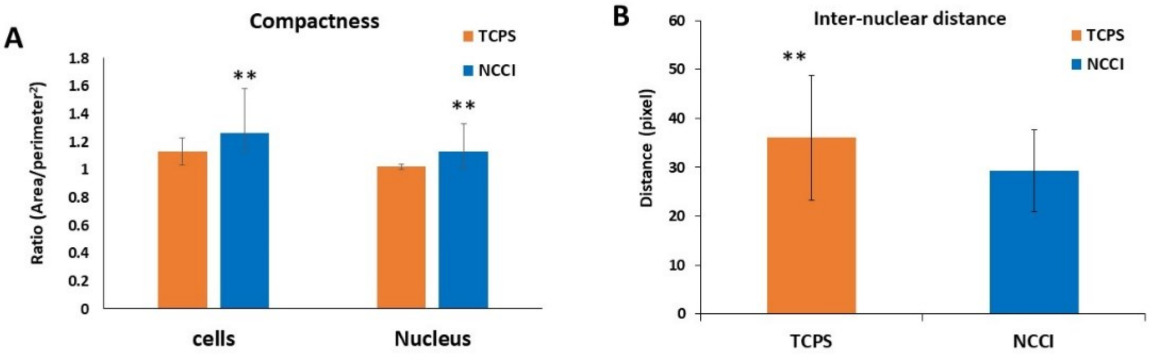

Figure S5: Graph showing the variation of **(A)** compactness and **(B)** Inter-nuclear distance for cells and nucleus grown on TCPS and NCCI .

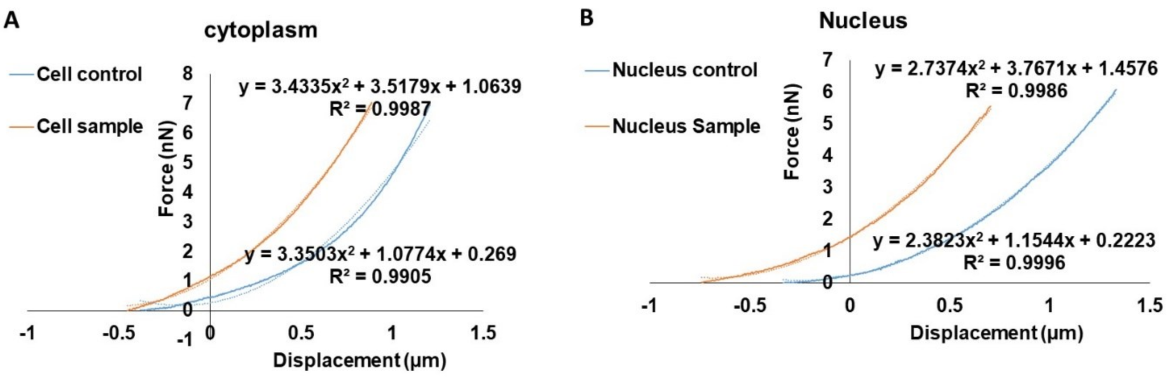

Figure S6: Graph showing force displacement curve during Nano indentation on **(A) c**ytoplasm **(B)** nucleus and **(C)** cell-to-cell junction on HaCaT cells grown on TCPS (control) and NCCI

Table S1: Bulk modulus (kPa) of cellular components for cells grown on TCPS and NCCI

Cell Component	TCPS	NCCI
Cytoplasm (n = 16)	5.06 ± 3.55	0.40 ± 0.23
Nucleus (n = 08)	1.06 ± 0.40	0.35 ± 0.23
Cell-to-cell junctions (n = 04)	2.75 ± 1.00	22.62 ± 18.37
